# Influence of Transgenic Metallothionein-1 on Gliosis, CA1 Neuronal Loss, and Brain Metal Levels of the Tg2576 Mouse Model of Alzheimer’s Disease

**DOI:** 10.3390/ijms18020251

**Published:** 2017-01-26

**Authors:** Gemma Comes, Yasmina Manso, Anna Escrig, Olaya Fernandez-Gayol, Paula Sanchis, Amalia Molinero, Mercedes Giralt, Javier Carrasco, Juan Hidalgo

**Affiliations:** Department of Cellular Biology, Physiology and Immunology, and Institute of Neurosciences, Universitat Autònoma de Barcelona, Bellaterra, 08193 Barcelona, Spain; gemma.comes@uab.cat (G.C.); ymanso.sanz@gmail.com (Y.M.); Anna.Escrig@uab.cat (A.E.); Olaya.Fernandez@uab.cat (O.F.-G.); Paula.Sanchis@uab.cat (P.S.); amalia.molinero@uab.cat (A.M.); merce.giralt@uab.cat (M.G.); javier.Carrasco@uab.cat (J.C.)

**Keywords:** Alzheimer’s disease, Tg2576, metallothionein-1, amyloid plaques, gliosis, CA1 neuronal loss, metals

## Abstract

The mouse model of Alzheimer’s disease (AD), Tg2576 mice (APP), has provided valuable information, such as the role of the metallothionein (MT) family in their behavioral and amyloidosis phenotypes. In this study, we further characterize the role of MT-1 by crossing *Mt1*-overexpressing mice with Tg2576 mice (APPTgMT). In 14-month-old mice, MT-1(/2) protein levels were dramatically increased by *Mt1* overexpression throughout the cortex (Cx), which showed a prominent caudal-rostral gradient, and the hippocampus (HC). There was a trend for MT-1(/2) immunostaining to be increased in the areas surrounding the amyloid plaques in control male mice but not in *Mt1*-overexpressing mice. Gliosis was elicited by the amyloid plaques, but the effects of *Mt1* overexpression were modest. However, in hippocampal western blots the microglial marker Iba-1 was increased in old male APPTgMT mice compared to APP-wild type (APPWT) mice, and the opposite was observed in young mice. Hippocampal CA1 neuronal loss was observed in Tg2576 mice, but was unaffected by *Mt1* overexpression. Aging increased Zn and Cu levels differently depending on brain area, sex, and genotype. Thus, the effects of *Mt1* overexpression on the phenotype of Tg2576 mice here studied are modest.

## 1. Introduction

Alzheimer’s disease (AD) is a devastating disease that causes a progressive loss of cognitive functions. It is characterized by the presence of extracellular deposits of amyloid-β peptides (amyloid plaques), intracellular deposits of hyperphosphorylated tau protein (neurofibrillary tangles), neuroinflammation, and oxidative stress in brain areas such as the hippocampus and the cortex [1,2]. Transition metals such as Cu or Fe, together with Zn, contribute to oxidative stress as well as to the aggregation and precipitation of amyloid-β peptides in the AD brain [3,4].

A number of studies suggest that the metallothionein (MT) family of proteins may be important for the understanding of AD. MT-1/2 isoforms have been shown to be upregulated in AD [5,6,7,8], whereas the results for MT-3 are less consistent [9,10]. In accordance with the human disease, MT-1/2 protein levels are increased in areas enriched in amyloid plaques in several AD mouse models, including the Tg2576 mice [8,11]. Ascertaining the putative role(s) of MTs in these mouse models is of great interest. Results obtained in transgenic mice crossing the Tg2576 mice with MT-1/2 deficient (*Mt1&2* KO) mice showed that these MT isoforms are involved in the formation of amyloid plaques, particularly in the hippocampus [12]. This possibility has been reinforced by recent results obtained crossing the Tg2576 mice with *Mt1*-overexpressing (TgMT) mice [13]. Moreover, some behavioral traits were also shown to be influenced by MT-1. Nevertheless, much remains to be understood. Here we expand the results by analyzing MT-1/2 immunohistochemistry, gliosis, neuronal survival, and Zn and Cu levels.

## 2. Results

### 2.1. MT-1/2 Immunostaining Is Dramatically Increased in TgMT Mice

**Figure 1:**
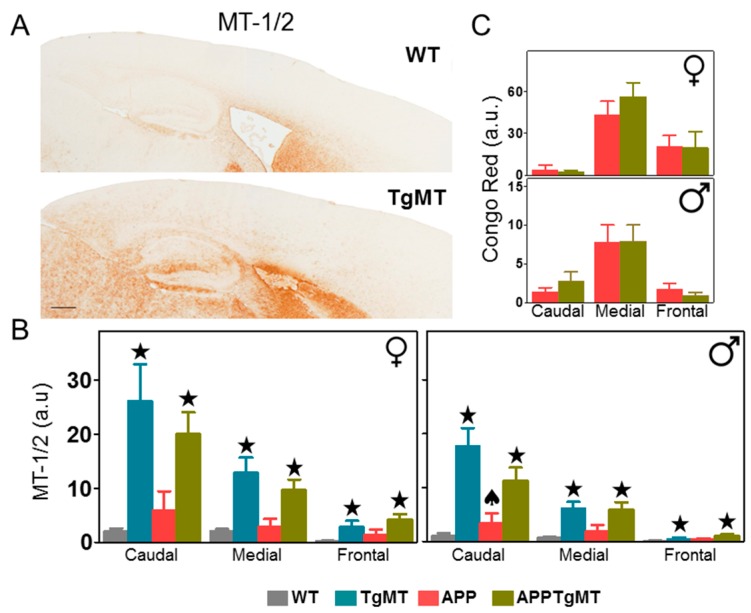
Effect of *Mt1* overexpression on MT-1/2 and Congo Red staining in the cortex. (**A**) Representative brain MT-1/2 immunostaining in wild-type (WT) (**top**) and TgMT (**bottom**) mice; (**B**) Quantification of MT-1/2 IHC of the different genotypes in the cortex showed a dramatic increase in *Mt1*-expressing (TgMT and APPTgMT) mice (★ *p* at least ≤0.05 vs. WT or APP mice, respectively) with a prominent caudal-frontal gradient. As revealed by the significant interaction between APP expression and *Mt1* overexpression (♠ *p* < 0.05 in male caudal region; the rest was not significant), APP expression tended towards an increase in MT-1/2 in WT mice; and the opposite was true in TgMT mice; (**C**) The greatest accumulation of dense amyloid plaques (stained with Congo Red) was localized in the medial area in both sexes. Results are mean ± SEM (*n* = 7–11). Scale bar: 400 µm. a.u., arbitrary units.

Representative MT-1/2 immunostaining in wild-type (WT) and TgMT mice (Figure 1A) as well as quantification of this staining in the cortex of the different genotypes (Figure 1B) clearly indicates that total MT-1/2 protein levels were dramatically increased throughout the brain in TgMT male and female mice (*p* < 0.001). The antibody used for MT immunohistochemistry (IHC) recognizes both MT-1 and MT-2 isoforms; the increase in total MT-1/2 levels presumably reflects the expression of the *Mt1* transgene (thus increasing MT-1 protein levels) rather than changes in MT-2 levels. Interestingly, we noticed a prominent gradient in MT-1/2 IHC, with the highest staining in the caudal cortex and the lowest in the frontal cortex (Figure 1B; *p* < 0.001). Such a gradient was present regardless of amyloid precursor protein (APP) expression, which produced a major accumulation of Congo Red positive dense amyloid plaques localized in the medial part of the brain but less so in the caudal and frontal cortex (Figure 1C; *p* < 0.001). MT-1/2 immunostaining showed a trend for increased levels in APPWT mice (compared to WT mice), whereas the opposite was observed in APPTgMT mice (compared to TgMT mice) (Figure 1B). In the cortex of male mice this resulted in a significant interaction between APP expression and *Mt1* overexpression (*p* < 0.05), and between APP expression, *Mt1* overexpression, and area of the cortex (*p* < 0.05).

**Figure 2:**
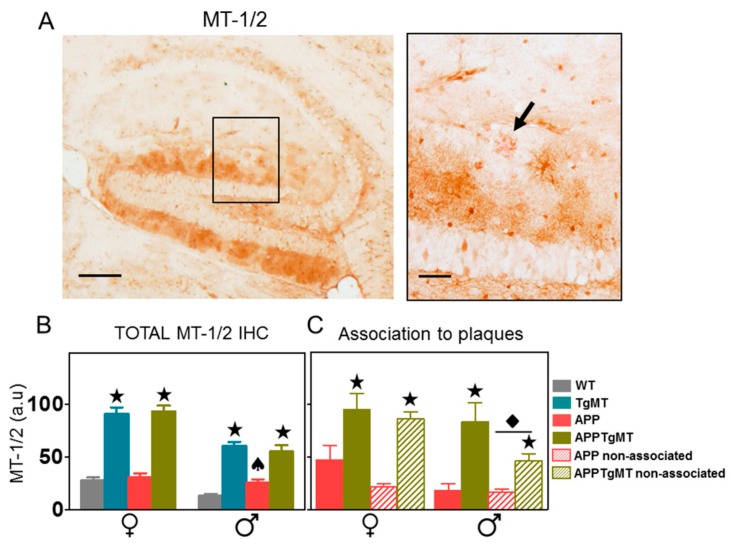
Effect of *Mt1* overexpression on MT-1/2 staining in the hippocampus. (**A**) Representative immunostaining for MT-1/2 counterstained with Congo Red in the hippocampus of APPTgMT mice (**left**); scale bar: 200 µm. A higher magnification of the black lined square area is shown at the right to better demonstrate plaques stained with Congo Red dye (**arrow**); scale bar: 50 µm; (**B**) Quantification of total MT-1/2 immunohistochemistry (IHC) produced similar results to the cortex, with dramatic increases in TgMT and APPTgMT mice (★ *p* < 0.001 vs. WT or APP mice, respectively). An opposing trend of APP expression was again seen between WT and TgMT male mice (♠ *p* < 0.05 interaction); (**C**) Comparison of MT-1/2 levels associated with plaques to those not associated with plaques indicated an increased immunostaining in the vicinity of the amyloid plaques only in male mice (◆ *p* < 0.05 vs. staining associated to plaques). Results are mean ± SEM (*n* = 7–11). a.u., arbitrary units.

In the hippocampus (Figure 2A,B), MT-1/2 immunostaining was also increased in TgMT and APPTgMT mice in both sexes relative to their respective controls (*p* < 0.001). As in the cortex, in the hippocampus of male mice the interaction between these two factors (APP and *Mt1* overexpression) was significant (*p* < 0.05), since there was an increase of MT-1/2 protein levels in APPWT mice compared to WT mice but not in APPTgMT mice compared to TgMT mice (Figure 2B). Double staining with Congo Red and MT-1/2 (Figure 2A) allowed comparisons of MT-1/2 protein levels in areas surrounding the dense amyloid plaques (Figure 2A right, arrow) to areas without plaques. As might be expected, there was a trend for increased MT-1/2 immunostaining near amyloid plaques, but this was significant (*p* < 0.05) only for male mice (Figure 2C). There were no significant differences in this regard in the cortex (data not shown). Thus, the effect of transgenic *Mt1* expression on MT-1/2 immunostaining is much more evident than changes in its association with amyloid plaques.

### 2.2. Mt1 Overexpression Has Only Minor Effects on the Gliosis Elicited by Amyloid Plaques

**Figure 3:**
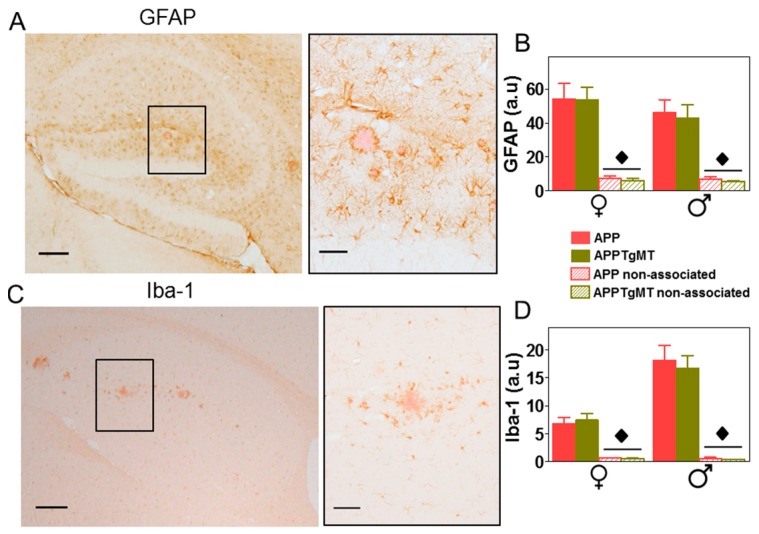
Effect of *Mt1* overexpression on gliosis in the hippocampus. (**A**,**C**) Representative immunostaining for GFAP (astrocytes) and Iba-1 (microglia), respectively, counterstained with Congo Red, in the hippocampus of APPTgMT mice (**left**); scale bar: 200 µm. On the right, a higher magnification of the black lined square area from left panel shows astroglia and microglia surrounding dense plaques; scale bar: 50 µm; (**B**,**D**) Quantification of GFAP and Iba-1 IHC indicated a dramatic increase in the vicinity of the plaques. Results are mean ± SEM (*n* = 11–18); ◆ *p* < 0.001 vs. plaque-associated staining. a.u., arbitrary units.

As expected, amyloid plaques elicited a dramatic gliosis in the hippocampus (Figure 3) and cortex (not shown). In contrast to MT-1/2 immunostaining, both GFAP (Glial Fibrillary Acidic Protein) (astrocytes; Figure 3A,B) and Iba-1 (Ionized calcium binding adaptor molecule 1) (microglia; Figure 3C,D) immunostainings were significantly (*p* < 0.001) increased in the area surrounding Congo Red-positive plaques compared to areas without plaques. *Mt1* overexpression did not appear to influence these immunostainings for astrogliosis and microgliosis (Figure 3B,D, respectively). Thus, the presence or absence of amyloid plaques appeared to influence gliosis in the hippocampus more than the expression of *Mt1*, at least in the tissue sections analyzed.

**Figure 4:**
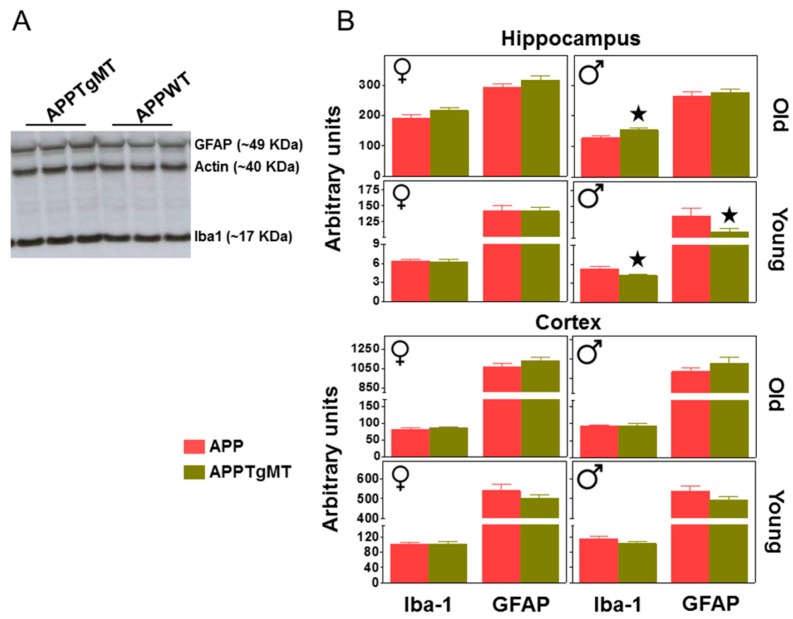
Effect of *Mt1* overexpression on hippocampal gliosis as measured by western blot (WB). Total hippocampal and cortex homogenates were assayed by WB to further characterize gliosis. (**A**) Representative band pattern of the WB (in an autoradiographic film) of old male hippocampus using antibodies for GFAP, Iba-1, and Actin; (**B**) Quantification of hippocampal GFAP and Iba-1 levels in young and old APPWT and APPTgMT mice. Iba-1 levels were increased by *Mt1* overexpression in old male mice but decreased in young males; the latter also showed decreased GFAP levels. Data are mean ± SEM (*n* = 10–11). ★ *p* at least ≤0.05 vs. APPWT mice. a.u., arbitrary units.

In contrast, results obtained by western blot using the whole hippocampus of one hemisphere did show small, but in some cases significant, effects of *Mt1* overexpression (Figure 4A,B). Thus, in the hippocampus of male mice a significant (*p* < 0.05) increase of Iba-1 levels was observed in old mice; the same trend was observed in female mice. In contrast, hippocampal Iba-1 levels were decreased by *Mt1* overexpression in young male mice (Figure 4B; *p* < 0.05)). GFAP levels followed a similar pattern. In contrast to the hippocampus, in the cortex, *Mt1* overexpression did not significantly influence gliosis at any age as evaluated by western blot (Figure 4B).

### 2.3. Mt1 Overexpression Does Not Affect Hippocampal CA1 Neuronal Loss

**Figure 5:**
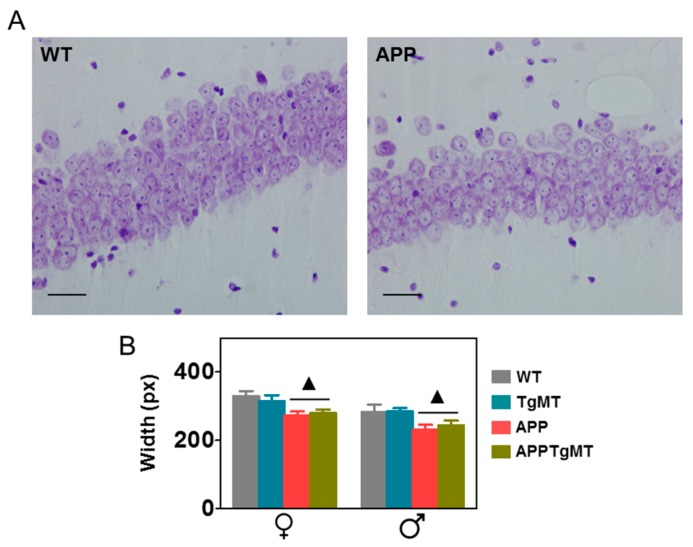
Effect of *Mt1* overexpression on hippocampal CA1 neurons. (**A**) Representative histochemistry of Nissl body staining of neurons in hippocampal CA1 of WT and APPWT mice. Scale bar: 20 μm; (**B**) Quantification of the thickness of the CA1 layer indicated a significant decrease in APPWT and APPTgMT mice in both sexes, whereas no significant effects of *Mt1* overexpression were observed. Results are mean ± SEM (*n* = 11–18); ▲ *p* < 0.01 vs. APP negative mice.

A clear neuronal loss was observed in the CA1 hippocampal area of Tg2576 male and female mice, with a clear thinning of the pyramidal layer as revealed by Nissl staining. This was not influenced significantly by *Mt1* overexpression (Figure 5).

### 2.4. Mt1 Overexpression Has only Minor Effects on Zinc and Copper Levels

**Figure 6:**
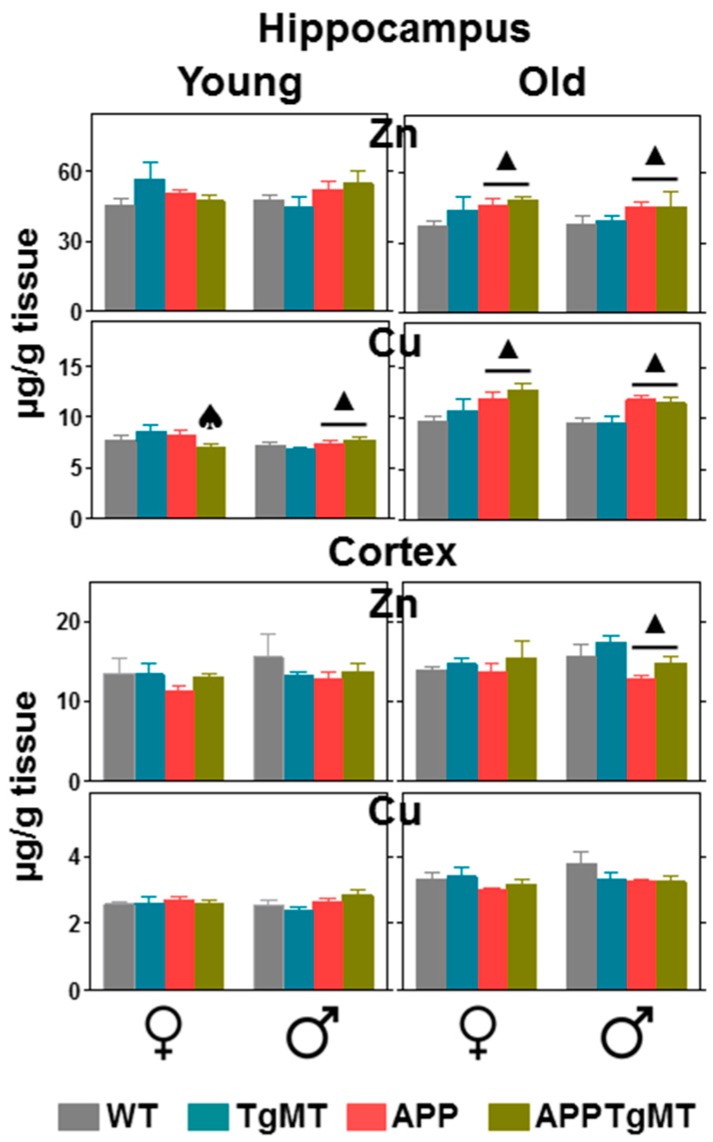
Effects of *Mt1* overexpression on Zn and Cu levels. Total hippocampal (**top**) and cortical (**bottom**) homogenates from young (~6 months) and old (~14 months) mice were analyzed by ICP-MS. In the hippocampus, copper and zinc levels were increased and decreased by aging, respectively; both metals were increased in the cortex. APP and *Mt1* expression showed different effects depending on the metal and brain area. Results are mean ± SEM (*n* = 7–11); ▲ *p* at least ≤0.05 vs. APP negative mice. ♠ *p* < 0.05 interaction between APP and TgMT.

Total hippocampal and cortical homogenates from young and old mice were used to assess zinc and copper content by ICP-MS (Figure 6). In the hippocampus, copper levels were increased by aging (*p* < 0.001), a trend favored by APP expression and in both sexes. *Mt1* overexpression decreased the effect of APP expression on copper levels of young female mice. Aging had a significant decreasing effect on zinc levels (*p* at least <0.05) in the hippocampus, but *Mt1* overexpression did not show a significant effect in this regard. In the cortex, both zinc and copper levels were moderately increased by aging (*p* at least <0.005); this trend was opposed by APP expression (significantly in male mice), in sharp contrast with the cortex. *Mt1* overexpression tended to increase cortex zinc levels, but variability precluded statistical significance.

## 3. Discussion

We previously showed that *Mt1* overexpression influenced the Tg2576 mice phenotype in a number of ways, including the formation of amyloid plaques and some behavioral traits [13]. It is important to emphasize that the effects caused by *Mt1* overexpression are consistent with those observed in Tg2576/*Mt1&2* KO mice [12]. For instance, *Mt1* overexpression slightly but significantly increased the amyloid load in the hippocampus, whereas the opposite trend was observed in *Mt1&2* KO mice. However, the mechanisms underlying these effects of MTs on amyloid plaques and other physiological/pathological variables remain to be fully determined. We herewith expand those results by analyzing gliosis, neuronal survival, and accumulation of essential metals in the critical brain areas for AD, cortex, and hippocampus.

Since amyloid plaques are mostly produced in the cortex and the hippocampus, we focused our analysis in these brain areas. We previously showed that *Mt1* (but not *Mt3*) mRNA levels measured by in situ hybridization were clearly increased in TgMT mice [13,14]. Since the antibody used recognizes both MT-1 and MT-2 isoforms, it was expected that MT-1/2 immunostaining would also be clearly increased, and indeed that was the case in both male and female mice. Interestingly, a caudal-frontal gradient in MT-1/2 IHC was observed in the cortex. It is likely that such a gradient could be related to the prominent expression of MT-1/2 in astrocytes [15,16,17], since these cells tend to show that type of gradient when expressing GFAP [18,19].

The caudal-frontal gradient in the MT-1/2 IHC was present regardless of the APP expression, although certainly some interesting trends could be observed. Thus, when comparing WT and APPWT mice, MT-1/2 immunostaining tended to increase in the latter, presumably because of the amyloid plaques and the associated neuroinflammation. This is in accordance with previous results with in situ hybridization, which indicated that *Mt1* mRNA levels were increased in cells surrounding the amyloid plaques [13,20]. The fact that the Tg2576 mouse model is an AD model with a relative paucity of amyloid plaques, added to the high basal expression of MT-1/2, makes it difficult to see prominent increases of these proteins, which nevertheless we observed, albeit only significantly in male mice. In contrast, when comparing TgMT and APPTgMT mice, MT-1/2 immunostaining tended to decrease in the latter. The very same pattern was observed in the hippocampus. The reason for these opposing trends remains to be established. They might be related to differences between the regulation of the normal (endogenous) MT-1/2 genes and that of the minimally marked *Mt1* (transgenic) gene. According to Palmiter et al. [21], the minimally marked *Mt1* gene, while being expressed about 50% less on a per gene basis, shows a normal tissue distribution, and responds normally to factors such as heavy metals, dexamethasone, and lipopolysaccharide (LPS), which in principle strongly suggests that the transgene is regulated in a similar fashion to the endogenous *Mt1* gene. Therefore, MT-1/2 immunostaining should also be increased by the amyloid plaques in the APPTgMT mice, but this did not occur. Thus, other reasons may be involved, perhaps specifically related to the amyloid plaques (rather than a general phenomenon such as stress) which deserves further attention.

Other putative mechanisms set in motion by *Mt1* overexpression could be related to altered gliosis and neuroinflammation and/or a modulation in normal metal ion homeostasis. MT-1/2 proteins have been shown to affect gliosis in a number of ways [14,16,17,22]. Astrocytes surrounding the amyloid plaques in old mice were more reactive as revealed by prominent GFAP immunostaining compared to areas without plaques, but, in accordance with our previous study using *Mt1&2* KO mice [12], *Mt1* overexpression did not significantly affect GFAP levels in either the hippocampus or cortex. Similarly, microgliosis, assessed with Iba-1 immunostaining, was not altered significantly by *Mt1* overexpression in the tissue sections of cortex and hippocampus that were analyzed. In contrast, results obtained by western blot using the whole hippocampus (and thus more representative compared to sampled sections) of one hemisphere of old male mice showed an increase of Iba-1 levels; the same trend was observed in female mice. This is consistent with the western blot results found in *Mt1&2* KO mice [12]. It is likely that this effect of *Mt1* overexpression is related to the increased amyloid plaque burden these mice show in the hippocampus [13]; in accordance, *Mt1&2* KO mice show decreased amyloid burden [12]. Thus, through an unknown mechanism, MT-1/2 seem to control the amyloid plaque deposition, which, in turn, drives microglial reactivity.

The situation is different at five months of age, since no amyloid plaques are yet present [23,24]. In contrast to the results found in old mice, *Mt1* overexpression significantly decreased both microgliosis and astrogliosis in the hippocampus; the same trend was present in the cortex. Remarkably, this was occurring again only in male mice. Moreover, the results are generally consistent with those found in *Mt1&2* KO mice [12]. An inhibitory effect of MT-1/2 on microglia has also been suggested in other studies [25,26,27]. Altogether, the present results suggest that while MT-1 may have a direct inhibitory role controlling microglia, it is overridden by an indirect stimulatory role in the case of APP positive mice because of its effects on the formation of amyloid plaques.

In old APP positive (APPWT and APPTgMT) mice, we readily observed neuronal loss of hippocampal CA1 neurons, a known hallmark of mice carrying the “Swedish mutation” [28]; interestingly, this is also observed in AD patients [29]. In this context, it was somewhat surprising that *Mt1* overexpression did not significantly influence neuronal survival [30,31,32]. Whether or not this is related to different neuronal susceptibilities, to MT-1 levels, or to the specific experimental model causing neurodegeneration remains to be established.

On the other hand, MTs are Zn, Cu-binding proteins [9], metals which have been reported to participate in amyloid-β peptide aggregation [33] and in ROS production [34]. As expected [12], aging, APP and *Mt1* overexpression affected metal content in a modest way. Yet, these were remarkable effects. Aging had different effects on Zn and Cu accumulation in the cortex and the hippocampus. Thus, in the cortex aging increased Zn (slightly) and Cu levels (more robustly), and this effect of aging was partially blunted in APP positive mice, which is consistent with previous studies [12,35]. In contrast, in the hippocampus, aging increased Cu levels but decreased Zn levels, and both Zn and Cu levels were increased by APP expression. These results highlight the importance of measuring metals in specific areas of the brain rather than bulk brains. Interestingly, increased copper and iron levels with aging have been proposed as a mechanism to explain the age-dependent onset of amyloid neuropathology in the same mice (Tg2576) [35], more so considering hippocampal neuropathology, where APP positive mice had an even higher accumulation of Cu with aging. *Mt1* overexpression only caused minor effects on metal levels, and thus they are unlikely to underlie the phenotype of APPTgMT mice in comparison to APPWT mice (see [12] for further discussion). It should be noted that we have measured total Zn and Cu levels, and therefore we cannot rule out specific effects of MT-1 on free metal ion levels and/or bound metal levels.

In summary, the present study evidences that while MT-1/2 are able to modulate the formation of amyloid plaques and some behavioral traits [12,13], MT-1 shows modest effects on glial activation, neuronal survival, and heavy metal accumulation.

## 4. Materials and Methods

### 4.1. Animals

The parental strains used in this study were C57BL/6JOlaHsd as a wild-type (WT) strain (Harlan, KY, USA), TgMT mice, which carry 56 copies of a minimally marked *Mt1* (Mt1*) gene [21] (B6.Cg-Tg(*Mt1*)174Bri/J; The Jackson Laboratory, Bar Harbor, ME, USA), and the AD mouse model Tg2576 which expresses the human APP_695_ harboring the Swedish K670N/M671L mutations under the control of the hamster prion protein promoter [23] (Taconic Europe A/S; Ry, Denmark). These strains were crossed and genotyped as previously described [13] to produce WT, TgMT, APPWT (APP in Figures), and APPTgMT mice. Throughout the manuscript, we may refer the two former groups as APP negative mice, and the two latter as APP positive mice. Mice were killed at ~6 and ~14 months of age.

Mice were housed in groups and given ad libitum access to food and water in a 12-h dark-light cycle under constant temperature (~22 °C). Animals were killed by decapitation and the brain quickly removed on placed ice. The cortex (Cx) and hippocampus (HC) of the right hemisphere were quickly dissected, frozen with liquid nitrogen, and stored at −80 °C. The left hemisphere was fixed by immersion in 4% paraformaldehyde and stored in 70% ethanol at 4 °C until further processing for paraffin-embedding. All experimental procedures were approved by the Ethics Committee in Human and Animal experimentation from the Autonomous University of Barcelona (CEEAH2996, 29 May 2015) and Servei de Biodiversitat i Protecció dels Animals (8837, 15 December 2015).

### 4.2. Immunohistochemistry (IHC) and Histochemistry (HC)

Fixed brains were paraffin-embedded and cut sagitally in 8 μm-thick sections for assessing MT-1/2 (primary antibody: anti-MT 1/100, DAKO, M0639, Clone 9; secondary antibody: biotinylated anti-mouse IgG 1:300, SIGMA, St. Louis, MO, USA), astrogliosis (primary antibody: anti-GFAP 1:900, DakoCytomation, Glostrup, Denmark A/S; secondary antibody: biotinylated anti-rabbit IgG 1:300, Vector Laboratories, Inc., Burlingame, CA, USA), and microgliosis (primary antibody: anti-Iba1 1:1500, WAKO, Tokyo, Japan; secondary antibody: biotininylated anti-rabbit IgG (H + L) 1:300, Vector Laboratories, Burlingame, CA, USA) as described [12].

All IHC performed were double-stained with Congo red stain (SIGMA) to identify areas with dense plaques with a congophilic core, in order to assess the MT-1/2 IHC and the activation of astrocytes and microglia surrounding the plaques compared to areas without plaques.

In order to quantify hippocampal CA1 neurons, 0.1% of Cresyl Violet (SIGMA) was used to stain Nissl substance. An image of CA1 of the hippocampus was acquired using a bright field microscope (Nikon Eclipse E400, Nikon Corporation, Tokyo, Japan). The images were analyzed using Image J software (1.49 v) [36] and the average of three measures of CA1 thickness was taken. Analyses were performed on two non-consecutive sections per mouse.

Stained sections were examined with a bright-field microscope (Nikon Eclipse 90i, Nikon Corporation) and images were acquired from the cortex and the hippocampus using a Nikon digital camera DXM 1200F and Nikon Act-1 v. 2.70 software. The images were analyzed using Image J software. A limited area was determined around the dense plaques stained with Congo red and the ImageJ color deconvolution plugin by Gabriel Landini [37] was used in order to separate the DAB and Congo red colors, obtaining afterwards the quantity of immunostaining associated to dense plaques, the quantity not associated and the total amount of staining of the brain areas studied (Cx and HC). The quantitation of immunostaining in the cortex was divided in three regions: caudal, medial (~above hippocampus) and frontal.

Histological analyses were performed on at least three non-consecutive sections per mouse.

### 4.3. Western Blotting

Total homogenates of Cx and HC were obtained by sonication in 50 mM Tris-HCl (pH 7.6), 0.01% NP-40, 150 mM NaCl, 2 mM EDTA, 3% sodium dodecyl sulfate (SDS), 1mM phenylmethylsulfonyl fluoride (PMSF), 1% sodium deoxycholate and protease inhibitor cocktail (Sigma-Aldrich, Madrid, Spain). Protein concentration was measured using the bicinchoninic acid (BCA) protein assay as specified by the manufacturer (Pierce, Thermo Fisher Scientific Inc; Rockford, IL, USA) and samples were stored at −80 °C until they were used. Western blot for astrocytosis (anti-Glial Fibrillary Acidic Protein –GFAP– 1:40,000, DakoCytomation, Denmark A/S) and microgliosis (anti-ionized calcium binding adaptor molecule 1 –Iba-1– 1:3000, Wako Pure Chemical industries, Osaka, Japan) was carried out as previously described [12]. Membranes were developed with ECL reagent (Amersham, GE Healthcare, Buckinghamshire, UK) and exposed to autoradiographic film (Kodak, Rochester, NY, USA); for quantification, images were acquired and quantified using the Bio-Rad laboratories (Hercules, CA, USA) QuantityOne ChemiDoc software (version 4.6.3).

### 4.4. Inductively Coupled Plasma-Mass Spectrometry (ICP-MS)

Cortical and hippocampal tissues were prepared as described above. Following digestion of the samples with HNO_3_ at 60 °C, and dilution in 1% HNO_3_, determination of Zn and Cu was carried out as described [12].

### 4.5. Statistical Analysis

Data was analyzed using the Statistical Package for Social Sciences (SPSS) version 17.0. Males and females were analyzed separately. The data was analyzed using Generalized Linear Model (GLZ) using APP (APP positive vs. APP negative) and *Mt1* overexpression (TgMT positive vs. TgMT negative) as main factors. In the IHC, when the intensity around the plaques was compared with the non-associated intensity, “association to plaques” was used as grouping factor; and when several areas of the cortex were studied, this was an additional factor. In the study of the metal content in the hippocampus “age” was used as a factor (young and old). Statistical significance was defined as *p* ≤ 0.05.
